# Analysis of euploidy rates in preimplantation genetic testing for aneuploidy cycles with progestin-primed versus GnRH agonist/antagonist protocol

**DOI:** 10.1186/s40001-023-01000-1

**Published:** 2023-01-16

**Authors:** Lu Wang, Jingyun Wang, Yuan Zhang, Chen Qian, Xiaohui Wang, Jie Bai, Fang Li, Zhiqin Chen, Ai Ai

**Affiliations:** 1 Shanghai Key Laboratory of Maternal Fetal Medicine, Shanghai Institute of Maternal-Fetal Medicine and Gynecologic Oncology, Shanghai First Maternity and Infant Hospital, School of Medicine, Tongji University, Shanghai, 200092 People’s Republic of China; 2 Department of Obstetrics and Gynecology, East Hospital, School of Medicine, Tongji University, No. 150 Jimo Road, Shanghai, 200120 People’s Republic of China

**Keywords:** Progestin-primed ovarian stimulation, Euploid blastocyst, Euploidy rate, Preimplantation genetic testing for aneuploidy, Live birth rate

## Abstract

**Background:**

Progestins can suppress endogenous luteinising hormone (LH) secretion from the pituitary gland and have shown similar efficacy in terms of collecting competent oocytes and embryos; however, some inconsistencies have been proposed regarding the quality of embryos collected with the use of progestins. This study aimed to evaluate euploidy rates and pregnancy outcomes in preimplantation genetic testing for aneuploidy (PGT-A) cycles using the progestin-primed ovarian stimulation (PPOS) protocol versus the gonadotropin-releasing hormone (GnRH) agonist/antagonist protocol.

**Methods:**

This retrospective cohort study included 608 PGT-A cycles: 146 women in the PPOS group, 160 women in the GnRH agonist group, and 302 women in the GnRH antagonist group. This study was performed at the in vitro fertilisation (IVF) centre of Shanghai First Maternity and Infant Hospital between January 2019 and December 2021. Additionally, 267 corresponding first frozen embryo transfer (FET) cycles were analysed to assess pregnancy outcomes.

**Results:**

The euploid blastocyst rate per injected metaphase II(MII) oocytes (14.60% vs. 14.09% vs. 13.94%) was comparable among the three groups (*p* > 0.05). No significant differences were observed among the three groups regarding pregnancy outcomes, including biochemical pregnancy, clinical pregnancy, ongoing pregnancy, implantation, miscarriage, ectopic pregnancy, and live birth rates per transfer in the first FET cycles (*p* > 0.05).

**Conclusions:**

The PPOS protocol had no negative effect on euploid blastocyst formation, and the pregnancy outcomes in FET cycles using the PPOS protocol were similar to those of the GnRH agonist and antagonist protocols.

*Trial registration* This trial was retrospectively registered

## Background

Ovarian stimulation is a crucial step in assisted reproductive technology (ART), aiming to collect multiple oocytes and generate a sufficient number of embryos available for transfer [1]. Conventional ovarian stimulation protocols include the gonadotropin-releasing hormone (GnRH) agonist protocol and GnRH antagonist protocol. However, these stimulation protocols have some disadvantages, such as increased procedure complexity, higher cost, greater risk of ovarian hyperstimulation syndrome (OHSS), and some patients experience a premature luteinising hormone (LH) surge [2].

A new ovarian stimulation protocol named progestin-primed ovarian stimulation (PPOS) was proposed by Kuang et al. in 2015 [3]. In this new protocol, progestin is used to suppress a premature LH surge during the follicular phase, thereby preventing premature ovulation. Medroxyprogesterone acetate (MPA), micronised progesterone, or dydrogesterone are administered orally instead of via repeated injections, which can reduce patient costs and discomfort. Recently, the clinical effectiveness and safety of PPOS have been demonstrated. Recent reports have suggested that the number of matured oocytes retrieved, and the resultant number of good-quality embryos using PPOS protocols are similar to that achieved using conventional protocols [4–6]. Nevertheless, debates on the benefits and challenges of PPOS versus GnRH agonist or antagonist protocols have been raised intensively. A recent randomised control trial showed that ovarian stimulation using MPA for the prevention of LH surge yielded a similar number of metaphase II (MII) oocytes as the GnRH antagonist in oocyte donation cycles, whereas the reproductive outcomes were unexpectedly poorer in recipients of oocytes in the MPA group. Additionally, another randomised control trial demonstrated that the number of MII oocytes, maturity rate, number of two pronuclei (2PN), and serum oestradiol levels on trigger day were statistically lower in the PPOS group than in the antagonist group; furthermore, PPOS did not improve biochemical and clinical pregnancy rates of infertile women, suggesting a possible impairment of oocyte competence when using MPA with freeze-all embryos [7].

Euploidy rate is an important indicator of embryo quality, and recent studies have demonstrated that the chance of having a live birth is mainly determined by the chromosomal status of the embryos [8]. Preimplantation genetic testing for aneuploidy (PGT-A) has been widely used for patients with advanced maternal age, recurrent miscarriage, or repeated implantation failure to avoid infertility or miscarriage caused by chromosomal abnormalities of the embryos [9]. Nagaoka et al. suggested that higher aneuploidy rates might be characterised by different ovarian stimulation regimens [10]. Moreover, Sato et al. proposed that ovarian stimulation interfered in the natural selection of dominant follicles, increasing the errors in the division of oocytes and genomic imprinting [11]. Hence, the principal goal of ovarian stimulation is to increase the chances of obtaining more euploid embryos [12]. In another randomised controlled trial, the GnRH antagonist protocol resulted in a higher rate of euploid embryos than the GnRH agonist protocol [13]. It has been demonstrated that elevated serum progesterone does not affect the number of euploid and good-quality embryos for transfer in GnRH antagonist cycles [14]; whereas, the impact of PPOS on the rate of euploid embryos remains unclear.

Therefore, this study aimed to explore whether PPOS has a negative effect on euploid blastocyst formation. We compared the rates of euploid embryos and pregnancy outcomes in frozen embryo transfer (FET) cycles using the PPOS protocol with FET cycles using the traditional protocols, including the GnRH agonist and antagonist protocols.

## Methods

### Study participants

This retrospective cohort study was performed at the Department of Assisted Reproduction of Shanghai First Maternity and Infant Hospital between January 2019 and December 2021. Women undergoing their first ovarian stimulation cycle with different indications for PGT-A were included if they met the following inclusion criteria: (i) advanced maternal age (≥ 38 years old); (ii) recurrent miscarriage (≥ 2 or 3 consecutive miscarriages), or (iii) repeated implantation failure (≥ 4 embryos transferred or ≥ 2 blastocysts transferred without success) [15]. They were excluded because of the following: (1) maternal or paternal monogenic disease or chromosomal abnormalities; (2) recipient of oocyte donation; and (3) presence of hydrosalpinx or endometrial polyp which was not surgically treated.

### Ovarian stimulation

The women started PGT-A with ovarian stimulation using either PPOS or agonist/antagonist protocols. For the PPOS protocol, dydrogesterone (20 mg/day, Abbott Biologicals B.V., the Netherlands) was administered from the day of ovarian stimulation until the day of ovulation trigger. For the GnRH agonist protocol, gonadotropin-releasing hormone analogue (GnRHa) (1.25 mg or 1.88 mg, triptorelin acetate, Diphereline, Ipsen Pharma Biotech, France) was administered for pituitary desensitisation from the mid-luteal phase in the previous cycle. On day 2‒3 of the menstrual cycle, patients underwent serum oestradiol measurement and transvaginal ultrasound examination. Recombinant follicle stimulating hormone (FSH) (Puregon, Organon, Dublin, Ireland or Gonal F, Merck Serono S.p.A, Modugno, Italy) or human menopausal gonadotropin (HMG) (Lizhu Pharmaceutical Trading Co., Zhuhai, China) was administered at 150‒225 IU per day based on the woman’s age, antral follicle count (AFC), and previous ovarian response, according to the standard operation procedures of the centre. Ovarian response was monitored using serial transvaginal scanning with hormonal monitoring. Further dosage adjustments were based on ovarian response at the discretion of the clinicians in charge. For the antagonist protocol, ganirelix (0.25 mg/day, Orgalutran, Organon, Dublin, Ireland) was administered from the fifth or sixth day of ovarian stimulation until the day of ovulation trigger.

When three leading follicles reached ≥ 18 mm in diameter, triptorelin (0.1 mg; Decapeptyl, Ferring Pharmaceuticals, Netherlands) and human chorionic gonadotropin (hCG 2000 IU or 5000 IU; Lizhu Pharmaceutical Trading Co., China) were administered to trigger final maturation of oocytes. Oocyte retrieval was performed approximately 36 h later.

### Fertilisation, embryo evaluation, and blastocyst culture

Semen samples were prepared using the swim-up procedure. Approximately 2 h following oocyte retrieval, fertilisation was performed via intracytoplasmic sperm injection. Oocytes were decoronated and checked for the presence of two pronuclei to confirm fertilisation. All embryos were cultured in the appropriate atmosphere until they reached the blastocyst stage. Blastocysts were graded according to the Gardner standard [16].

### Preimplantation genetic testing for aneuploidy

For patients undergoing PGT-A, trophectoderm (TE) biopsy was performed on good-quality blastocysts, and approximately five cells were aspirated gently and separated from the blastocyst by applying multiple pulses of a non-contact 1.48-μm diode laser (Saturn 5 ActiveTM, Cooper Surgical, Inc., CT, USA) through a zona pellucida opening created by the laser. The biopsied cells were subsequently washed three times in 1× phosphate buffered saline (PBS) (Life Technologies, NY, USA), transferred to a polymerase chain reaction (PCR) tube containing 2.5 μl 1× PBS, and cryopreserved at – 80 °C until analysis was performed. The samples were analysed and interpreted in genetic laboratories. Genetic screening was performed using next-generation sequencing (NGS)-based VeriSeq PGS assay following standard protocols and manufacturer recommendations (Illumina Inc., San Diego, USA). The PGT-A report classified embryos as euploid, aneuploid, mosaic, or non-conclusive. Euploid embryos were transferred, whereas aneuploid and mosaic embryos were not.

### Cryopreservation and FET

All good-quality blastocysts after TE biopsy were cryopreserved using vitrification. After PGT-A, the patients underwent FET if they had at least one euploid frozen blastocyst.

Vitrification was performed with MediCult Vitrification Cooling (Origio, Denmark), using ethylene glycol, propylene glycol, and sucrose as cryoprotectants. For the warming procedure following vitrification, the straw was cut and the capillary was pulled from the straw out of the liquid nitrogen and immediately warmed one by one using MediCult Vitrification Warming (Origio, Denmark). After warming, embryos were transferred into a culture dish and eventually to the uterus.

FETs were performed in natural cycles for ovulatory women and in clomiphene-induced or hormonal cycles for anovulatory women. Only one euploid blastocyst was transferred during FET cycles.

### Follow-up

A urine pregnancy test was performed 2 weeks after the transfer. Those with a positive urine pregnancy test were scanned after 2 weeks to identify the number and presence of a gestational sac with a foetal pole. All pregnant women were contacted or traced for pregnancy outcomes after delivery or miscarriages.

### Outcomes measures

The primary outcome measure was the euploid blastocyst rate, defined as the number of euploid embryos per injected MII. Secondary outcome measures included the euploid blastocyst rate per injected MII, biochemical pregnancy rate, clinical pregnancy rate per transfer/per woman, implantation rate, ongoing pregnancy rate, miscarriage rate, ectopic pregnancy rate, and live birth rate per transfer/per woman in FET cycles. Clinical pregnancy was defined as the presence of at least one gestational sac on ultrasonography at 6 weeks. The implantation rate was calculated as the number of gestational sacs observed on scanning divided by the number of embryos transferred. Ongoing pregnancy was defined as the presence of at least one foetus with heart pulsation on ultrasound after 10 weeks. A baby born alive after 22 weeks of gestation was classified as a live birth. The miscarriage rate was defined as the number of miscarriages before 22 weeks divided by the number of women with biochemical pregnancies. The ectopic pregnancy rate was defined as the number of ectopic pregnancies before 22 weeks divided by the number of women with biochemical pregnancies.

### Statistical analyses

Continuous variables were presented as medians (interquartile range), while categorical data were presented as percentages. Statistical comparisons were performed using Kruskal‒Wallis test for continuous variables and the Chi-square test for categorical data. Post hoc analysis for individual group comparisons was performed if the overall *p*-value was < 0.05. A multivariate linear regression model was used to evaluate the association between the euploid blastocyst rate and the use of ovarian stimulation protocols, adjusted for potential correlations. Statistical analysis was performed using the Statistical Program for Social Sciences (SPSS Inc., version 26.0, Chicago, USA) software. Statistical significance was set at *p* < 0.05. In post hoc testing, the *p*-value was considered significant when < 0.017 [Bonferroni correction (0.05/3)].

## Results

### Participant flow

**Fig. 1:**
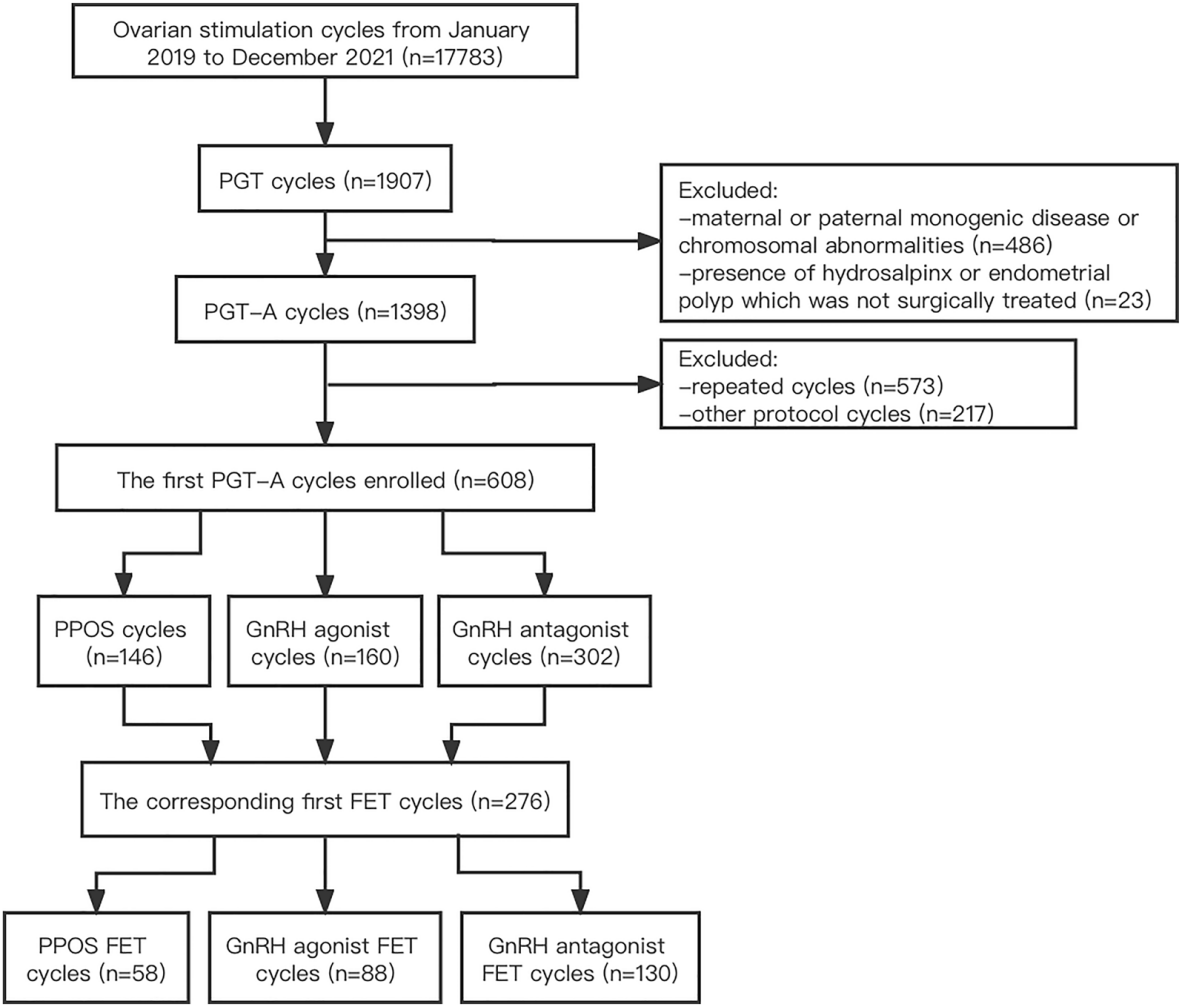
Flowchart of the study

Continuous variables from 17,783 cycles conducted between January 2019 and December 2021 were screened. Overall, 10,029 women did not meet the selection criteria; therefore, 608 women who underwent their first PGT-A cycle were included. Ovarian stimulation was performed in 146 women in the PPOS group, 160 women in the agonist group, and 302 women in the antagonist group. During the study period, 58 women in the PPOS group, 88 in the agonist group, and 130 in the antagonist group completed their first FET cycles (Fig. 1).

### Baseline characteristics

The baseline characteristics of the patients in the three groups are summarised in Table 1. No significant differences were observed in terms of infertility duration, anti-Müllerian hormone (AMH), and AFC (*p* > 0.05). However, female age, basal FSH level (bFSH), body mass index (BMI), type of infertility, and indication for PGT-A differed among the three groups (*p* < 0.05).

**Table 1:** Demographic characteristics of the cycles

Variables	Ovarian stimulation protocol			*p* value
	PPOS	GnRH agonist	GnRH antagonist	
No. of PGT-A cycles	146	160	302	
Female age (years)	38(33–41)	35(32–39)	37(33–40)	0.001^a,c^
BMI (kg/m^2^)	22.05(20.3–24.2)	21.6(20.3–23.4)	21.2(19.6–23.2)	0.010^b^
Primary infertility (%)*	22.6%(33/146)	11.25%(18/160)	21.52%(65/302)	0.013^a,c^
Secondary infertility (%)*	77.4%(113/146)	88.75%(142/160)	78.48%(237/302)	0.013^c^
Infertility duration (years)	1.5(1–3.75)	2(1–4)	2(1–3)	0.896
AMH	2.49(1.12–5.59)	3.33(2.33–4.76)	2.7(1.45–4.86)	0.06
bFSH	7.59(5.6–9)	6.3(4.98–7.28)	7.11(5.97–8.52)	0.000^a,c^
AFC	12(6–20)	14(10–19)	11(6–17)	0.138
Indication of PGT-A (%)*				0.001
Advanced maternal age	26.03%(38/146)	14.38%(23/160)	24.83%(75/302)	
Recurrent miscarriage	19.18%(28/146)	44.38%(71/160)	32.78%(99/302)	
Repeated implantation failure	25.34%(37/146)	19.38%(31/160)	16.89%(51/302)	
Mixed	29.45%(43/146)	21.88%(35/160)	25.50%(77/302)	

^a^PPOS vs. GnRH agonist, *p* < 0.05

^b^PPOS vs. GnRH antagonist, *p* < 0.05

^c^GnRH agonist vs. GnRH antagonist, *p* < 0.05

*P-value is considered to be significant when < 0.016 [Bonferroni correction (0.05/3)]

### Characteristics and outcomes of ovarian stimulation

There were no significant differences in progesterone levels on trigger day, cleavage rate, blastocyst formation rate, and number of euploid blastocysts. The euploid blastocyst rate per biopsy (46.54% vs. 46.04% vs. 45.78%), euploid blastocyst rate per injected MII (14.60% vs. 14.09% vs. 13.94%), and the number of cycles with no transferable blastocysts did not differ significantly among the three groups (*p* > 0.05).

Moreover, the duration of stimulation was shorter and the total dose of gonadotropin (Gn) was smaller in the PPOS group. However, the serum LH level, MII rate, and fertilisation rate were higher in the PPOS group than in the other two groups. The number of retrieved MII oocytes, fertilised oocytes, and blastocysts formed were lower, whereas the number of cycles with no blastocyst formation was higher in the PPOS group than in the GnRH agonist group (similar to that in the antagonist group) (Table 2).

**Table 2:** Characteristics and outcomes of ovarian stimulation

Variables	Ovarian stimulation protocol			*p* value
	PPOS	GnRH agonist	GnRH antagonist	
No. of PGT-A cycles	146	160	302	
Total dosage of Gn (IU)	1800(1575–2025)	2550(2071.86–3150)	2025(1593.75–2400)	0.000^a,b,c^
Duration of stimulation (days)	8(7–9)	11.5(10–13)	9(8–9)	0.000^a,b,c^
Oestradiol level on triggering day (pg/ml)	2420.92(1290–3533.49)	2641(1973.32–3668.44)	2017.36(1268.5–3017)	0.003^c^
Serum LH level on trigger day (IU/l)	3(1.94–5)	0.86(0.59–1.68)	2(1–3.29)	0.000^a,b,c^
Progesterone level on trigger day (ng/ml)	1(0.77–1)	0.98(0.71–1.26)	1(0.73–1.32)	0.588
Total No. of retrieved oocytes	1280	1892	2999	
No. of retrieved oocytes	7(4–12)	12(7–15)	8(5–13)	0.000^a,c^
Total No. of MII	1062	1483	2374	
No. of MII (*n*)	6(3–10)	9(6–12)	6(4–11)	0.000^a,c^
MII rate (%)	82.97%(1062/1280)	78.38%(1483/1892)	79.16%(2374/2999)	0.004^a,b^
Total No. of oocytes fertilised (*n*)	815	1014	1716	
No. of oocytes fertilised (*n*)	4.5(2–8)	6(3–9)	5(3–8)	0.014^a,c^
Oocytes fertilised rate (%)*	76.74%(815/1062)	68.37%(1014/1483)	72.28(1716/2374)	0.000^a,b,c^
Total No. of cleaving embryos (*n*)	793	975	1668	
Cleavage rate (%)*	97.3%(793/815)	96.15%(975/1014)	97.2%(1668/1716)	0.24
Total No. of blastocyst culture	765	996	1625	
Total No. of blastocysts formation	333	454	723	
No. of blastocysts formation (*n*)	2(0–3)	2(1–4)	2(1–4)	0.036^a,c^
Blastocysts formation rate (%)*	43.53%(333/765)	45.58%(454/996)	44.49%(723/1625)	0.687
Total No. of euploid blastocysts	155	209	331	
No. of euploid blastocysts (*n*)	0(0–2)	1(0–2)	1(0–2)	0.084
Euploid blastocysts rate per biopsy (%)*	46.54%(155/333)	46.04%(209/454)	45.78%(331/723)	0.973
Euploid blastocysts rate per injected MII (%)*	14.60%(155/1062)	14.09%(209/1483)	13.94%(331/2374)	0.878
No. of PGT cycle with no blastocyst (%)	28.1%(41/146)	14.38%(23/160)	21.52%(65/302)	0.013^a^
No. of PGT cycle with no transferable blastocysts (%)*	51.37%(75/146)	38.75%(62/160)	47.02%(142/302)	0.074

^a^PPOS vs. GnRH agonist, *p* < 0.05

^b^PPOS vs. GnRH antagonist, *p* < 0.05

^c^GnRH agonist vs. GnRH antagonist, *p* < 0.05

*p-value is considered to be significant when < 0.016 [Bonferroni correction (0.05/3)]

### Pregnancy outcomes

A total of 276 first FET cycles with only one euploid blastocyst were performed. Although endometrial preparation and endometrial thickness on the transfer day were significantly different (*p* < 0.05), no significant differences were observed among the three groups in all pregnancy outcomes, including biochemical pregnancy, clinical pregnancy, ongoing pregnancy, and live birth rates for the first FET cycles. Moreover, the implantation, miscarriage, and ectopic pregnancy rates were also not significantly different among the three groups (Table 3, *p* > 0.05).

**Table 3:** Comparison of pregnancy outcomes

Variables	Ovarian stimulation protocol			*p* value
	PPOS	GnRH agonist	GnRH antagonist	
Patients (*n*)	58	88	130	
Endometrial preparation, (%)*				
Natural cycles	8.62%(5/58)	38.64%(34/88)	20%(26/130)	0000^a^^,c^
Clomid-induced	17.24%(10/58)	9.09%(8/88)	7.69%(10/130)	0.124
Hormonal cycles	75.86%(44/58)	52.27%(46/88)	72.31%(94/130)	0.002^a,c^
Endometrial thickness (mm)	9.6(8.63–10.6)	8(8–9.75)	9(8–10)	0.001^a^
Biochemical pregnancy rate per transfer (%)*	70.69%(41/58)	60.23%(53/88)	60.77%(79/130)	0.364
Clinical pregnancy rate per transfer (%)*	51.72%(30/58)	57.95%(51/88)	56.15%(73/130)	0.755
Implantation rate (%)*	51.72%(30/58)	57.95%(51/88)	56.15%(73/130)	0.755
Ongoing pregnancy rate per transfer (%)*	46.55%(27/58)	52.27%(46/88)	50%(65/130)	0.795
Miscarriage rate (%)*	7.32%(3/41)	9.43%(5/53)	12.66%(10/79)	0.637
Ectopic pregnancy rate (%)*	4.88%(2/41)	0.000	1.27%(1/79)	0.181
Live birth rate per transfer (%)*	43.1%(25/58)	45.45%(40/88)	39.23%(51/130)	0.648
Cumulative clinical pregnancy rate per woman (%)*	28.08%(41/146)	32.5%(52/160)	26.49%(80/302)	0.393
Cumulative live birth rate per woman (%)*	20.55%(30/146)	27.5%(44/160)	19.21%(58/302)	0.112

^a^PPOS vs. GnRH agonist, *p* < 0.05

^b^PPOS vs. GnRH antagonist, *p* < 0.05

^c^GnRH agonist vs. GnRH antagonist, *p* < 0.05

*p-value is considered to be significant when < 0.016 [Bonferroni correction (0.05/3)]

During the study period, 58 women in the PPOS group, 88 in the agonist group, and 130 in the antagonist group underwent 75, 86, and 122 FET cycles, respectively. The women had at most four completed FET cycles. The cumulative clinical pregnancy rate and live birth rate per woman were similar among the three groups.

### Multivariate linear regression

The multivariate linear regression model using the enter method with variables including the woman’s age, BMI, duration of infertility, type of infertility, AMH, bFSH, ovarian stimulation protocol, serum oestradiol, LH, progesterone levels on trigger day, total dosage of Gn, duration of stimulation, and total number of retrieved oocytes showed that only the woman’s age and duration of infertility, but not ovarian stimulation protocol (*p* = 0.852), were associated with the euploidy rate of blastocysts in the PGT-A cycles (Table 4).

**Table 4:** Multivariate liner regression analysis for euploidy rate

Independent variable	β	*t*	*p*-value	95% CI for Exp(B)	
				Lower	Upper
Age	− 0.455	− 7.543	0.000	− 4.184	− 2.451
BMI	− 0.04	− 0.756	0.45	− 1.955	0.87
Duration of infertility	− 0.12	− 2.06	0.04	− 2.974	− 0.067
Type of infertility	0.028	0.487	0.627	− 8.332	13.802
AMH	0.001	0.019	0.985	− 1.254	1.279
bFSH	− 0.047	− 0.897	0.371	− 1.459	0.546
AFC	0.081	1.552	0.122	− 0.106	0.892
Indication of PGT-A	− 0.069	− 0.49	0.624	− 25.518	15.348
Ovarian stimulation protocol	0.027	0.187	0.852	− 18.883	22.839
Oestradiol level on triggering day (pg/ml)	− 0.006	− 0.109	0.914	− 0.002	0.002
Serum LH level on trigger day (IU/l)	− 0.014	− 0.265	0.791	− 0.017	0.013
Progesterone level on trigger day (ng/ml)	− 0.022	− 0.416	0.678	− 2.545	1.658
Total dosage of Gn (IU)	0.083	1.057	0.291	− 0.003	0.01
Duration of stimulation(days)	− 0.044	− 0.515	0.607	− 2.688	1.574
Total number of retrieved oocytes	0.006	0.041	0.967	− 1.651	1.721

## Discussion

In this study, we demonstrated identical euploidy rates of blastocysts in PGT-A cycles using three different ovarian stimulation protocols. Moreover, pregnancy outcomes in the FET cycles showed similar results.

Our results indicated that progestins were capable of effectively preventing premature ovulation in PGT-A cycles; however, the LH level on hCG day was significantly lower in the agonist and antagonist groups, indicating that progesterone can be used as an alternative to GnRH agonist/antagonist for suppressing premature LH surge during ovarian stimulation in IVF cycles but the effect was weaker compared to that with the antagonist. We also observed that the total gonadotropin dose was lower, and the day of stimulation was shorter in the PPOS group than in the long GnRH agonist and antagonist groups. This may be due to the prolonged pituitary suppression in the long-agonist protocol which started from the mid-luteal phase of the previous cycle; moreover, prolonged pituitary downregulation by GnRHa might contribute to improving endometrial receptivity [17].

In this study, we found that the number of oocytes obtained, fertilised oocytes, cleaving embryos, and transferable embryos were lower in the PPOS group than in the long-agonist group. These results are in contrast to previous studies that showed comparable embryological characteristics in progestin and short GnRH agonist cycles [18–21]. Studies with FET cycles provide an opportunity to assess different protocols for oocyte quality and subsequent embryo development potential. In the first FET cycles, we observed similar clinical pregnancy and live birth rates per FET as well as implantation rates in the PPOS group compared to those in the other two groups, indicating that the embryos originating from the PPOS protocol may have similar development potential to those from the agonist and antagonist protocols. Furthermore, we combined several FET cycles from each individual woman during the study period and concluded that the cumulative clinical pregnancy and live birth rates per woman were also comparable among the three groups.

Our study demonstrated that the total number of blastocysts, number of euploid blastocysts, and euploidy rate were similar between PPOS and conventional stimulation protocols. This result indicates that PPOS might have no impact on embryo quality, at least when assessed by analysing the chromosomes of the embryo. Our results were consistent with those of La Marca et al. [22], which demonstrated that the rate of euploid formation per injected oocyte was similar in patients undergoing PPOS and in patients undergoing the GnRH antagonist protocol. However, only 48 patients were recruited in the PPOS group of that study, which claimed an age-matched historical case–control study rather than a cohort study according to their study design. Notably, pregnancy outcomes after FET were not reported. Further studies are needed on long-term obstetric outcomes before this protocol can be introduced on a large scale.

According to Ata’s study, PPOS combined with an elective freeze-all approach may not be currently justified for all IVF cycles because avoiding a fresh transfer does not seem beneficial in the absence of a medical indication when a fresh embryo transfer is not intended [23, 24]. In PPOS, total freezing of the obtained embryos and delayed transfer are mandatory. In cases where fresh embryo transfer is not required, such as fertility preservation, oocyte donation, or PGT, PPOS may be recommended and proposed as a first-line treatment [25]. Therefore, the potential harmful effects of the hormonal environment on endometrial receptivity are avoided. Other patients who can benefit from this protocol are those at risk of OHSS, because, for these patients, the application of the “freeze-all” strategy and triggering can be exerted by the GnRH agonist, which helps to avoid early-onset OHSS.

Our study is limited because of its retrospective and nonrandomised design. Some imbalanced characteristics were found in this study, and a multiple linear regression analysis was performed to control the biases. Cancellation or postponement of FET was different in the three groups; some patients did not undergo one FET but had frozen euploid blastocysts. Therefore, these results should be interpreted with caution. Further large randomised trials with adequate sample sizes are required to confirm these findings.

## Conclusions

Our findings revealed that the PPOS protocol did not have a negative effect on euploid blastocyst formation, and that pregnancy outcomes in FET cycles from PPOS were similar to those of the GnRH agonist and antagonist protocols.

## Data Availability

All data generated or analysed during this study are included in this published article.

## Abbreviations

ART: Assisted reproductive technology
GnRH: Gonadotropin-releasing hormone
OHSS: Ovarian hyperstimulation syndrome
LH: Luteinising hormone
PPOS: Progestin-primed ovarian stimulation
MPA: Medroxyprogesterone acetate
MII: Metaphase II
2PN: Number of two pronuclei
PGT-A: Preimplantation genetic testing for aneuploidy
FET: Frozen embryo transfer
GnRHa: Gonadotropin-releasing hormone analogue
FSH: Follicle stimulating hormone
HMG: Human menopausal gonadotropin
AFC: Antral follicle count
PBS: Phosphate buffered saline
hCG: Human chorionic gonadotropin
TE: Trophectoderm
PCR: Polymerase chain reaction
NGS: Next-generation sequencing
SPSS: Statistical Package for the Social Science
AMH: Anti-Müllerian hormone
bFSH: Basal follicle stimulating hormone
BMI: Body mass index
Gn: Gonadotropin
